# Supplementary material for: Epigenetic modifications affect the rate of spontaneous mutations in a pathogenic fungus
Source: Nat Commun. 2021 Oct 7;12:5869. doi: 10.1038/s41467-021-26108-y (PMC8497519; doi:10.1038/s41467-021-26108-y)
Supplement: Supplementary file 1 — Supplementary Information [file 41467_2021_26108_MOESM1_ESM.pdf]

Supplementary Information for:

Epigenetic modifications affect the rate of spontaneous mutations in a pathogenic fungus

Michael Habig[1,2*], Cecile Lorrain[1,2], Alice Feurtey[1,2], Jovan Komluski[1,2] and Eva H. Stukenbrock[1,2*]

Supplementary Methods 2
Supplementary Figure 11 2
Supplementary Figures 8
Supplementary Figure 1 8
Supplementary Figure 2 9
Supplementary Figure 3 10
Supplementary Figure 4 11
Supplementary Figure 5 12
Supplementary Figure 6 14
Supplementary Figure 7 15
Supplementary Figure 8 16
Supplementary Figure 9 18
Supplementary Figure 10 20
Supplementary Tables 22
Supplementary Table 1 22
Supplementary Table 2 23
Supplementary Table 3 24
Supplementary Table 4 25
Supplementary References 26

SUPPLEMENTARY METHODS

Mutation accumulation experiment

A single colony derived directly from a plated dilution of glycerol stock of the indicated *Zymoseptoria tritici* strain was resuspended in 1 mL YMS including 25% glycerol by 2 min vortexing on a VXR basic Vibrax at 2000 rpm, and 10–50 µL were re-plated onto a YMS agar plate. For each strain we generated and propagated 40 replicate lines. Cells were grown for 7 days at 18°C or 28°C until a random colony (based on vicinity to a prefixed position on the plate) derived from a single cell was picked and transferred to a new plate as described above. The transfers were conducted for one year (18°C, 52 times) or 4 weeks (28°C, 4 times) before the DNA of a randomly chosen colony of each replicate was extracted and sequenced (Supplementary Fig. 11).

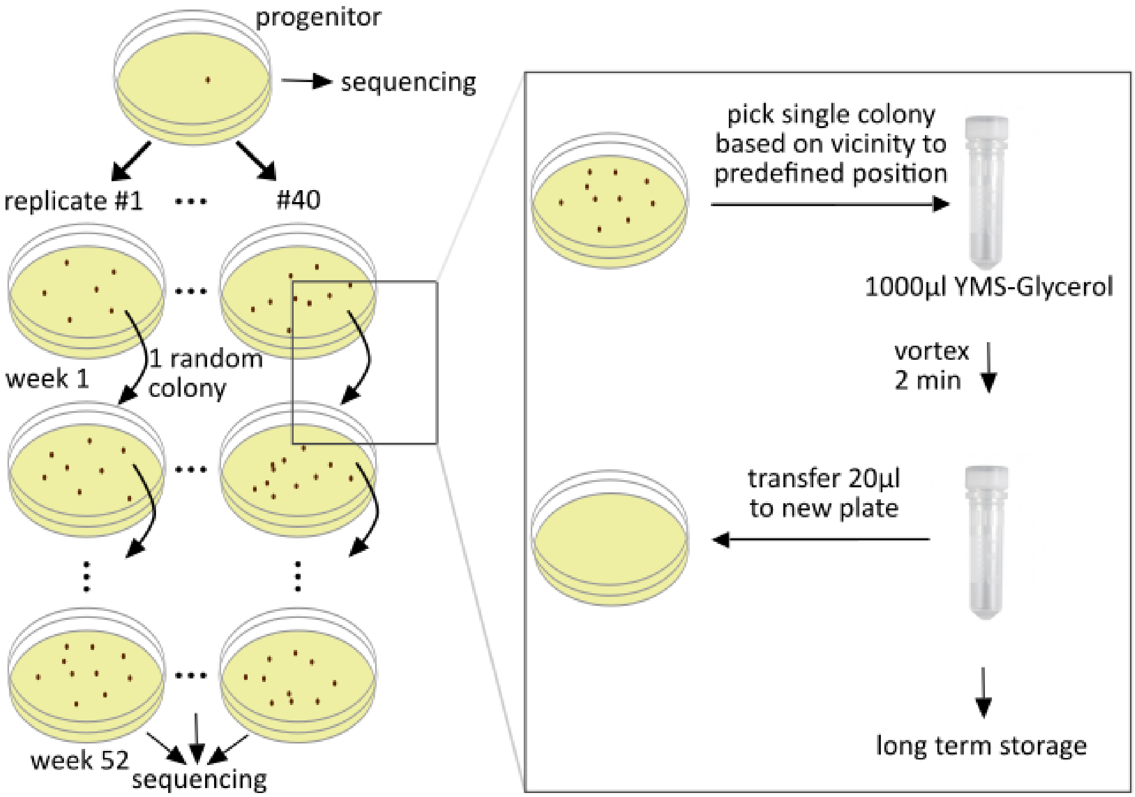

Supplementary Figure 11**. Experimental set-up for mutation accumulation experiment, exemplified for one strain.**

Mutation accumulation experiments maximize drift and minimize selection. Therefore, any adaptation (e.g. to different environmental conditions) should not occur. Hence mutation rates can be compared between different treatments by normalizing with the total number of cell divisions and the number of mutable sites. (see below).

The deletion mutants of *kmt1* and *kmt6* were generated in an IPO323 derivative that had lost chromosome 18 (IPO323 Δchr18) and this derivate was included in the mutation accumulation experiment. Therefore, all data of the deletion mutants (IPO323 Δchr18 Δ*kmt1* and IPO323 Δchr18 Δ*kmt6*) was compared to the IPO323 Δchr18 data only. In addition, we included the IPO323 isolate in order to be able to make comparison with the Zt05 and Zt10 isolates. Therefore, all data of Zt05 and Zt10 was compared to the IPO323 data only. We also analyzed correlations between histone modifications and mutation rates in the wildtype. For these correlations the data of IPO323 and IPO323 Δchr18 was pooled in order to increase the statistical power and resolution of comparisons between different genomic regions.

DNA isolation and Genome sequencing

For sequencing DNA of 286 strains (6 progenitor and 280 evolved replicated MA lines) was isolated using a standard protocol [1]. In short, cells were grown in 5 mL liquid YMS media for 5-7 days at 18°C in a shaking incubator at 200 rpm and harvested by centrifugation (10 min, 2000 x g). DNA was isolated from the pellet by adding 500 µl lysis buffer (10% Triton X100, 1% SDS, 100 mM NaCl, 10 mM EDTA, 10mM TrisHCL, pH8.0) and 500 µl phenol/chloroform (1/1 [v/v]) and approx. 100µl glass beads (Satorious, Göttingen, Germany) and shaken for 30 min at 2000 rpm (IKA Vibrax, IKA, Staufen, Germany) followed by centrifugation for 15 min at 17,000 x g. The upper phase was transferred to 1000 µl of Ethanol and the precipitate collected by centrifugation for 5 min at 17,000 x g. The supernatant was removed, and the pellet resolved in 50 µl TE-buffer including 10 µg/mL RNase A and incubated for 10min at 50°C. DNA integrity was checked via gel electrophoresis and concentration determined using Qubit fluorometer (ThermoFisher, Dreieich, Germany). Genome sequencing was conducted at the Max Planck-Genome-centre, Cologne, Germany using an Illumina HiSeq2500 machine and Covaris fragmentation during library preparation for 2 x250 bp paired end reads.

Determination of base substitutions, small INDELs and structural variations

Paired-end reads of 250 bp were mapped to the genome of the reference isolate IPO323 [2] (Accession: GCA_000219625.1) , Zt05 [3,4] or Zt10 [3,4] and processing of the reads was carried out using the below listed pipeline:

1) Quality filtering using Trimmomatic V0.38 [5]

```
java -jar /trimmomatic-0.30.jar PE R1.fastq R2.fastq R1_paired.fastq R1_unpaired.fastq R2_paired.fastq R2_unpaired.fastq ILLUMINACLIP:TruSeq3-PE.fa:2:30:10 LEADING:25 SLIDINGWINDOW:4:25 AVGQUAL:25 MINLEN:50
```

2) Mapping to reference genome using Bowtie 2 version 2.3.5 [6] and samtools version 1.7

```
bowtie2 --sensitive -x reference.fasta -1 R1_paired.fastq -2 R2_paired.fastq |
samtools view -q10 -bS |
samtools sort –o sorted.bam
```
http://bowtie-bio.sourceforge.net/bowtie2/index.shtml

3) Marking of duplicate reads, adding readgroup information and resorting using Picard version 2.18.20

```
java -jar picard.jar AddOrReplaceReadGroups INPUT= sorted.bam \
OUTPUT=sorted_RG.bam \
RGID=sample_ID RGLB=lib1 \
RGPL=ILLUMINA RGPU=unknown \
RGSM=sample_ID SORT_ORDER=coordinate
java -jar picard.jar MarkDuplicates I= sorted_RG.bam \
O=sorted_RG_Dedup.bam M=sorted_RG_Dedup.txt
```

4) SNP calling using samtools (version1.7) and bcftools (version 1.6)

```
samtools mpileup -E -C50 -Q20 -q20 -uf IPO323_reference R.bam | bcftools
call --ploidy-file ploidy.txt -vc -O u -o raw.bcf
```

5) Filtering of SNPs using bcftools (version 1.6)

```
bcftools filter -o Q20DP8AF08.bcf -e'QUAL<20 | DP<8 | AF<0.8' raw.vcf
```

6) Pairwise comparison to progenitor strain to output SNPs unique to the evolved replicates using bcftools (version1.6)

```
bcftools isec -c all -p sample Q20DP8AF08.bcf.gz ref.fasta >
private_to_progenitor.vcf
```

Determination of fungal growth rates

The progenitor strain IPO323Δchr18 and the replicated mutation accumulation (MA) lines that had been incubated at 28°C for 4 weeks, were also included in an *in vitro* phenotype assay under stress conditions as described in [7] and in an *in vitro* assay to determine the growth rate and the carrying capacity in liquid yeast malt sucrose (YMS) media. Hereby, 175µl of cell suspension at $1*10^4$ cells/ml in YMS were inoculated in 96 well plates at 18°C, 200 rpm for 231h and the optical densities obtained during this time were fitted using a logistic growth model with the growthrates (version 0.8.2) package in R. The initial doubling time for all progenitor strains was determined in three biological replicates by inoculating $5*10^5$ cells/mL in 25 mL YMS and incubating them at the specified conditions for 54h and determining the growth in cell number over this period (Supplementary Table 2).

Determination of mutation rates

The base substitution μ was calculated as described in [8]: $\mu = \frac{m}{\sum_1^n N \times T}$ with m the total number of all mutations in the respective genomic compartment across all MA lines, N the number of sites analyzed in each of the lines, T the total number of cell divisions for each of MA lines. Poisson distribution of count data was used to calculate the 95% confidence interval of the mutation rates for the genomic compartments analyzed. The equilibrium GC content was estimated according to [9] by $p = \frac{\mu_{A/T \to G/C}}{\mu_{A/T \to G/C} + \mu_{G/C \to A/T}}$ with the standard error of p being calculated as described in [10] by $SE(p) = \sqrt{\frac{p^2 \times \left(\frac{\sigma^2(u)}{\mu_u^2} - \frac{2\sigma(u,v)}{\mu_u \times \mu_v} + \frac{\sigma^2(v)}{\mu_v^2}\right)}{N}}$. The absolute rate of structural mutations was calculated accordingly. As all structural variations affect a length >1, the location within a specific genomic compartment was analyzed using the start and the end-positions for deletions, duplications, insertions and inversions, and additionally the position where an insertion occurred for all insertions.

Mutation rates were calculated per replicated MA line. The following genomic compartments were considered for all IPO323 derived strains: Histone modifications (H3K4me2, H3K9me3, H3K27me3) [11], Gene models [12],Transposable elements (TE) [3] and for all possible combinations of Transposable elements, H3K9me3 and H3K27me3.The size of the genomic compartment was adjusted for each replicate line by removing all chromosomes with a coverage of less than 5% of the average coverage. The average size of the genomic compartments for each of the genotype x environment combination and the respective number of SNPs are depicted in Supplementary Table 3.

Verification of mutations by Sanger Sequencing

For a total of 65 mutations (of which 51 passed the filter criteria for being included in the analysis and 14 did not pass the filter) a validation via Sanger sequencing was conducted. All of the 51 mutations that passed the filter criteria could be confirmed using Sanger sequencing while for all 14 that did not pass the filter criteria no mutation could be detected via Sanger sequencing (Supplementary Data 4). A list of all primers used in the study are listed in Supplementary Table 4.

Analysis of structural variations

Structural variations were identified by comparison of the evolved strains and the respective progenitor strains. Hereby Illumina reads were quality filtered as described above and mapped onto the reference genome using SpeedSeq align followed by structural variation analysis by LUMPY [13] as implemented in the SpeedSeq package (version 0.1.2) [14] using the following lines:

```
speedseq align -R '@RG\tID:id\tSM:samplename\tLB:lib' -t 10 -o mapped
ref.fasta forward.paired.fq reverse.paired.fq
speedseq sv -o compared_to_ref -R ref.fasta -g -m 1 \
                        -B mapped.bam \
                        -D mapped.discordants.bam \
                        -S mapped.splitteres.bam
```

The vcf files were filtered using bcftools version 1.6 as following: VCF files were filtered on genotype (GT=1/1 for deletions/insertions, GT=0/1 or GT=1/1 for duplications and inversions) and quality >25. Deletions larger than 70kb were disregarded.

To restrict the reported deletions to the novel ones in the evolved replicates we used bedtools intersect to remove all those deletions that overlap for >90% of their length with a structural variation in the respective progenitor strain using the following line:

```
bedtools intersect -v -header -F 0.9 -a evolved.filtered.vcf -b progenitoy-
filtered.vcf > unique.vcf
```

Due to low sensitivity of detecting insertions using LUMPY we chose an assembly-based approach for the detection of insertions in the evolved strains. We generated assemblies from quality filtered Illumina reads for all evolved replicated MA lines as following:

Merging of overlapping reads using PEAR (version 0.9.11) [15]

1) pear -f forward.paired.fq -r reverse.paired.fq. -j 8 -n 0 -k -o sample.ID

Generating assemblies using SPADES version (version 3.13.1) [16]

2) spades.py --pe1-1 unassembled.forward.fastq.gz --pe1-2

   unassembled.reverse.fastq.gz --pe1-m assembled.fastq.gz -m 96 --careful

   --threads 6 -o sample.ID

All contigs above a coverage of 15 were included in the subsequent analysis.

The reads of the progenitor strain were mapped on these assemblies as described above and structural variations were detected as described above by LUMPY [13] as implemented in the SpeedSeq package (version 0.1.2) [14]. The detected structural VCF files were filtered on genotype GT=1/1 and quality >25 and restricted to deletions. These deletions were considered insertions that occurred

in the replicated MA lines compared to the progenitor strain. The inserted sequence and up to 500 bp upstream and downstream of the insertions were extracted from the assemblies and mapped onto the respective progenitor genome using Bowtie 2 version 2.3.5 [6]:

```
bowtie2 --sensitive -x reference.fasta -f insert.fasta
bowtie2 --sensitive -x reference.fasta -f upstreamflank.fasta
bowtie2 --sensitive -x reference.fasta -f downstreamflank.fasta
```

For 29 of the total 154 inserts, no corresponding sequence in the respective reference genome was found - due to their low complexity - and these were therefore excluded from the analysis. For all others the location of the mapped insertions on the reference genomes was considered the origin of the insertion, whereas the mapped location of the upstream and downstream flanks on the reference genome were considered the destination of the inserts. For all 124 insertions that could be mapped onto the reference genome the corresponding upstream and downstream flanks were mapped adjacent to each other (Supplementary Data 5).

Validation of structural variation detection approach

To confirm the specificity and sensitivity of the detection of structural variation of the assembly-based approach we simulated a total of 820 random insertions and deletions in a total of 40 simulated genomes of the reference isolate genome IPO323. Randomly selected transposable elements were deleted or inserted at random positions within the genome. Reads were simulated from these genomes as well as from the IPO323 reference genomes using ART (v2.5.8) [17] with the following parameters: -l 250 -f 60 -m 477 -na -s 156 to simulate the characteristics of the reads obtained via sequencing. With these simulated reads and genomes, the insertions and deletions were detected as described above. The specificity of the detection was 100%, i.e. no additional and therefore false insertions and deletions were detected, whereas the sensitivity was at 86% (703 of the 820 insertions and deletions were detected) (Supplementary Data 6). The sensitivity for insertions and deletions larger than 50bp was at 92% (702 of 760 insertions and deletions).

SUPPLEMENTARY FIGURES

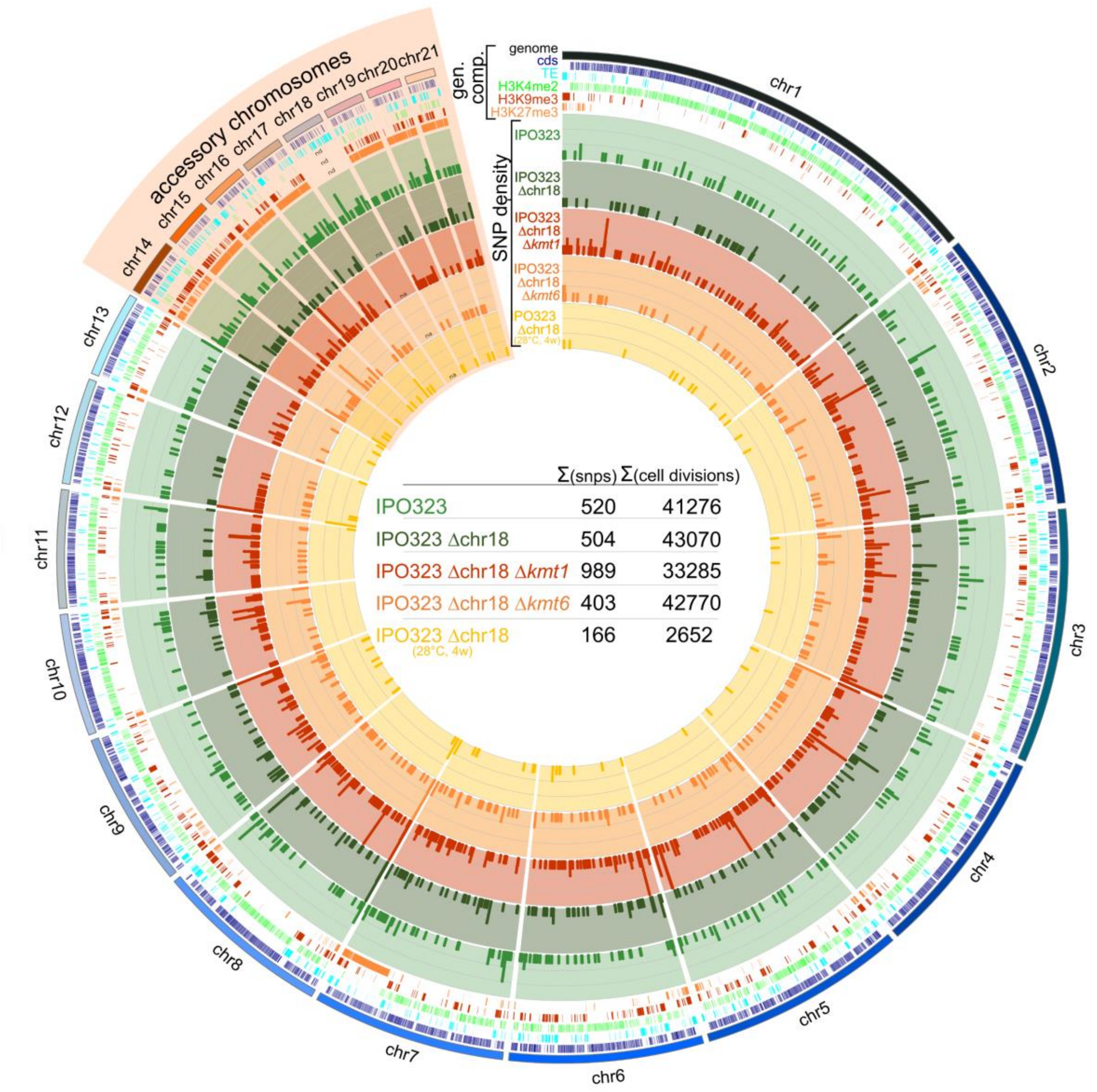

Supplementary Figure 1. **Circosplot of base substitution mutations in IPO323 derived strains.** Data depicted for IPO323 (light green) and IPO323 Δchr18 (dark green) and for deletion mutants of histone methyltransferase IPO323 Δchr18 Δ*kmt1* (red) and IPO323 Δchr18 Δ*kmt6* (orange) obtained after 52 weeks growth at 18°C and for IPO323 Δchr18 obtained after 4 weeks growth at 28°C (yellow). The density of base substitution mutations is depicted in 10 kb windows along 21 chromosomes of the IPO323 reference genome. In addition, the distribution of the chromosome compartments (coding sequences (cds, dark blue) transposable elements (TEs, aquamarine), H3K4me3 (green), H3K9me3 (red) and H3K27me3 (orange) are displayed.

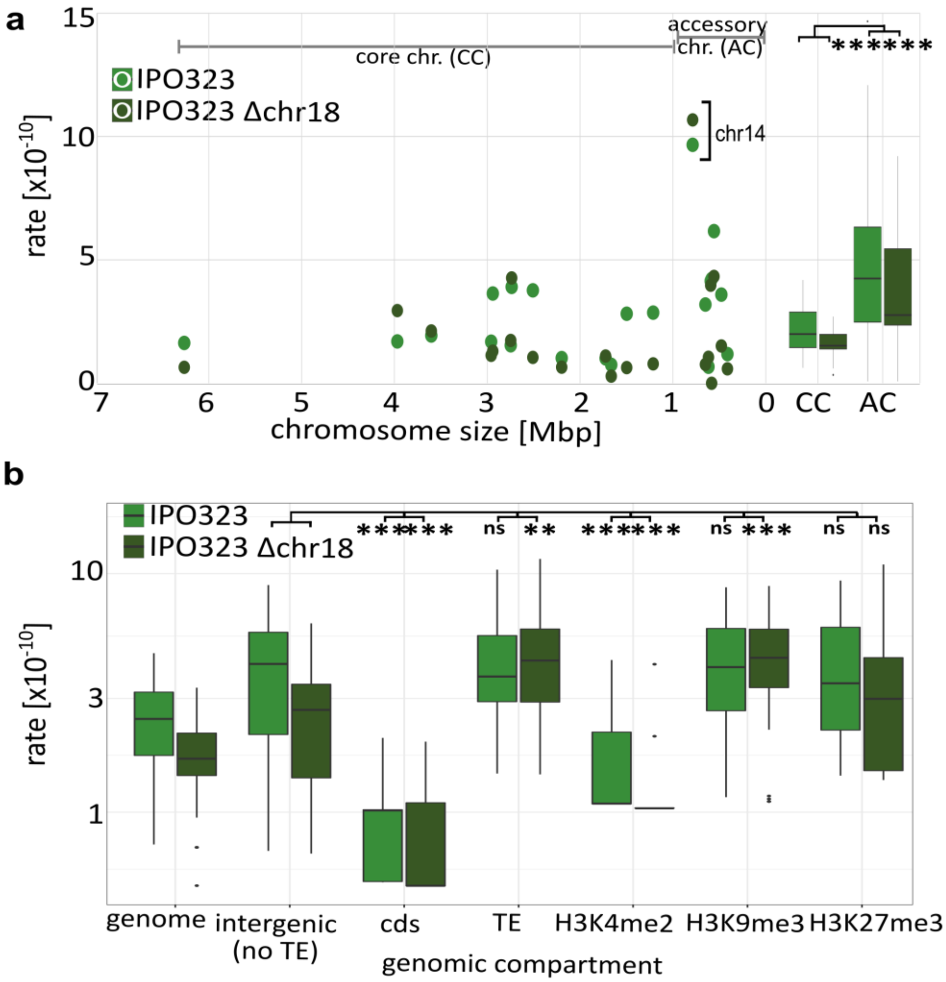

Supplementary Figure 2. **The rate of INDELs varies between different genomic compartments.** Comparison of the rates for small INDEL mutations between different genomic compartments. a) Correlation between the mean rate for the occurrence of INDELs (per site per cell division) for each of the chromosomes for both IPO323 (light green) and IPO323 Δchr18 (dark green) after 52 weeks at 18°C, also including a boxplot comparing the rate INDEL occurred within all replicated MA lines between core chromosomes (CC) and accessory chromosomes (AC) (n=40 independently evolved MA lines for each strain). b) Boxplot comparing the INDEL rate in the indicated genomic compartments within each replicated MA line of the IPO323 and IPO323 Δchr18 (n=40 independently evolved MA lines for each strain). Categorized FDR-corrected p-values of a & b) two-sided paired Wilcoxon test are shown (*: $p<0.05$, **: $p<0.005$, ***: $p<0.0005$). The exact p-values for all pairwise comparisons are provided in Supplementary Data 1. Box plots depict center line, median; box limits, upper and lower quartiles; whiskers, 1.5x interquartile range; points, outliers.

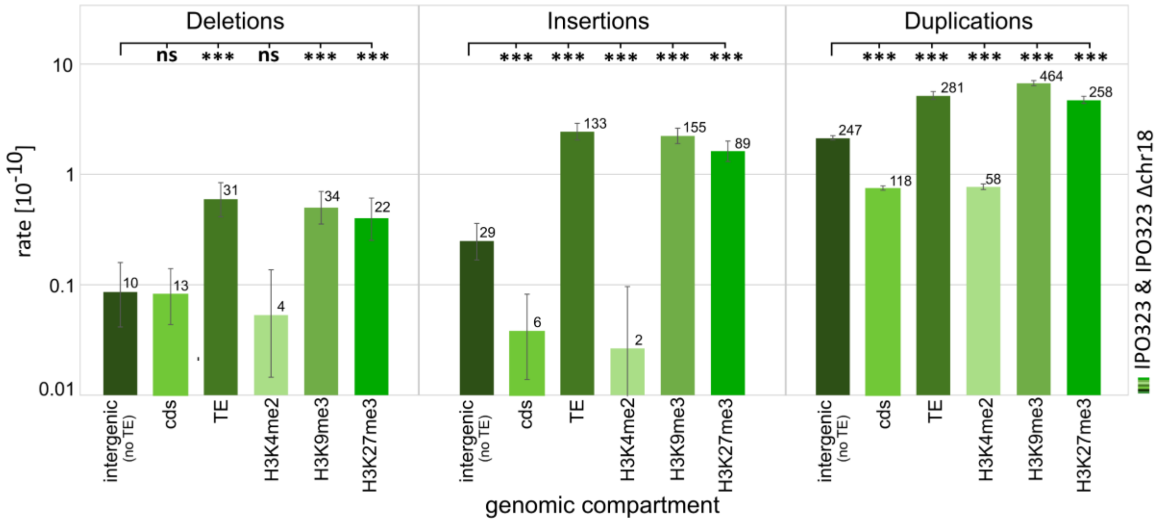

Supplementary Figure 3. **Rate of occurrence of the start and end positions of the structural variants in different genomic compartments** observed in IPO323 and IPO323 Δchr18 after 52 weeks growth at 18°C. Data was pooled for all replicated MA lines of IPO323 and IPO323 Δchr18 (n=80 independently MA evolved lines) and is presented as mean values. Categorized p-values of two-sided $\chi 2$ test corrected by FDR for multiple testing are shown. (*:p<0.05, **: p<0.005, ***:p<0.0005). The exact p-values for all pairwise comparisons are provided in Supplementary Data 1. Error-bars represent 95% Poisson confidence intervals. The total number of observed structural variation positions is depicted above each bar.

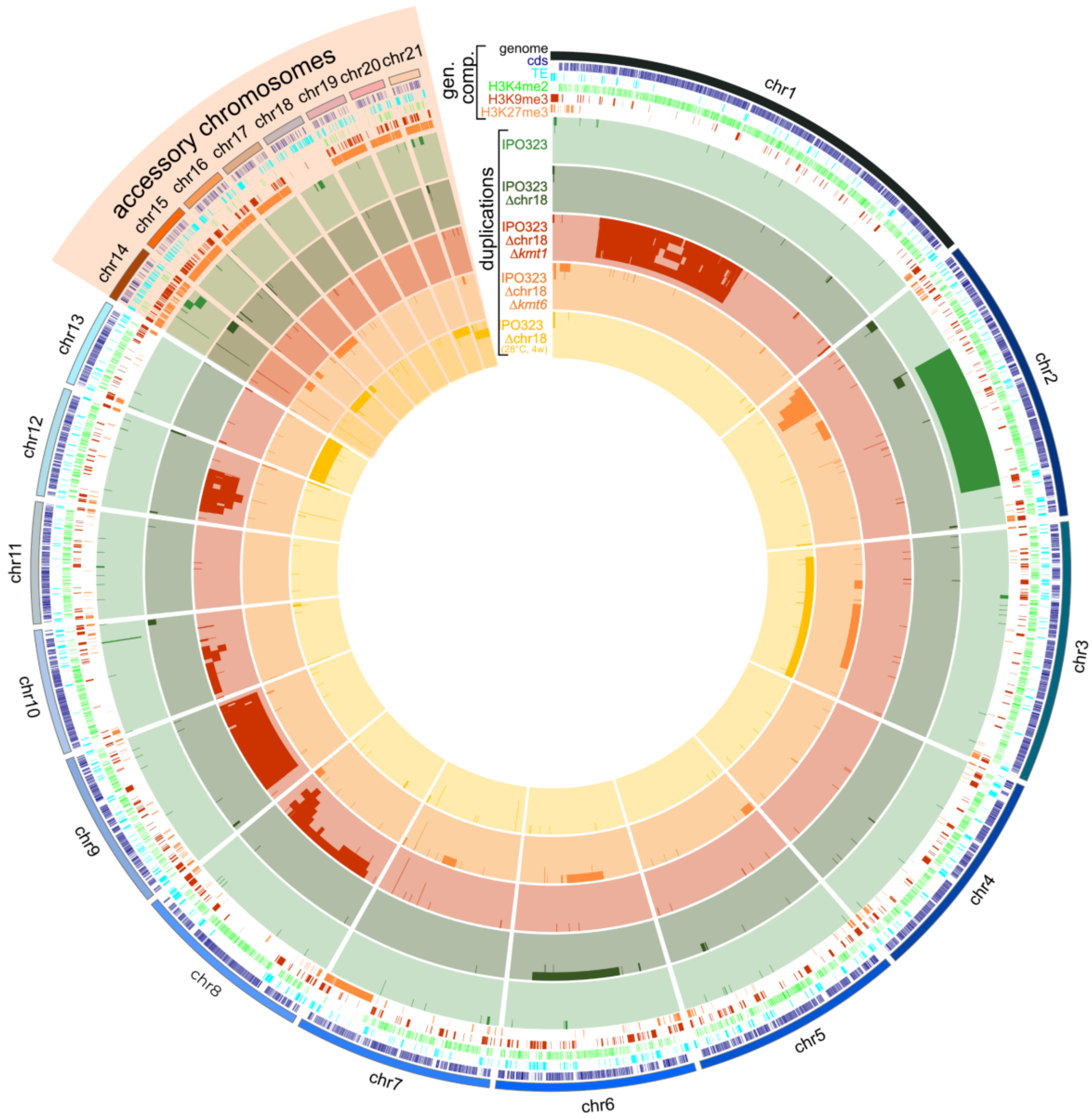

Supplementary Figure 4. **Circosplot of the locations of duplications in IPO323 derived strains.** Data depicted for IPO323 (light green) and IPO323 Δchr18 (dark green) and for deletion mutants of histone methyltransferase IPO323 Δchr18 Δ*kmt1* (red) and IPO323 Δchr18 Δ*kmt6* (orange) obtained after 52 weeks growth at 18°C and for IPO323 Δchr18 obtained after 4 weeks growth at 28°C (yellow). In addition, the locations of cds, TE and the post-translational histone marks H3K4me2, H3K9me3 and H3K27me3 are given.

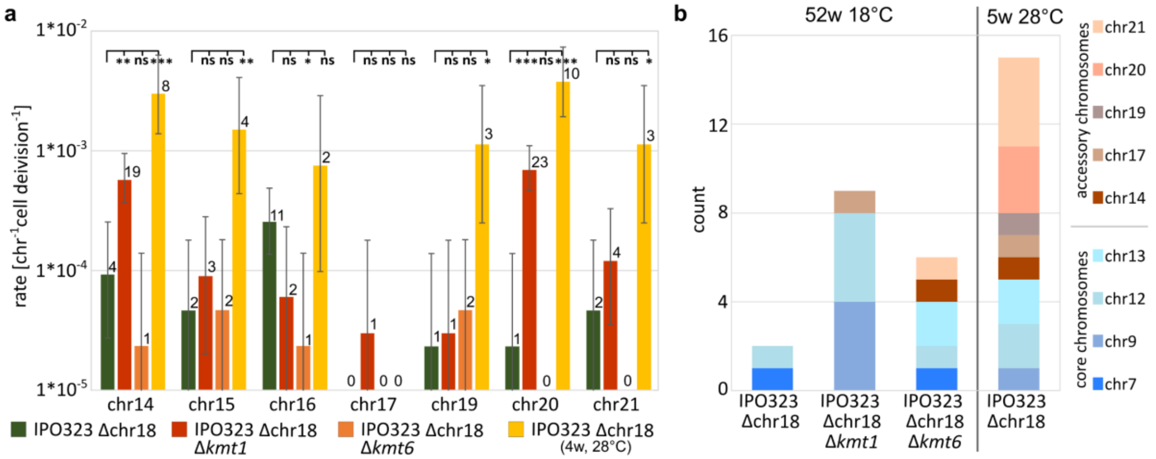

Supplementary Figure 5. **Effect of removal of histone modifications and increased temperature on the transmission of chromosomes during mitosis.** a) The rate of losses of distinct accessory chromosomes is affected differently by the removal of histone modifications and the increase in temperature. Bar chart comparing the rate of chromosome losses for all replicated MA lines of IPO323 Δchr18 and IPO323 Δchr18 Δ*kmt1* (lacking H3K9me3) and IPO323 Δchr18 Δ*kmt6* (lacking H3K27me3), propagated at 18°C for 52 weeks, and IPO323 Δchr18 propagated for 4 weeks at 28°C (n=40 independently evolved MA lines per strain). b) Duplication rate for entire core and accessory chromosomes according to genotype and temperature. Duplicated core chromosomes are depicted in blue hues and duplicated accessory chromosomes are depicted in brown hues. a) Data is presented as mean values. Error bars represent 95% Poisson confidence intervals. Categorized p-values of FDR adjusted two-sided Fisher's exact test are depicted (*:$p<0.05$, **: $p<0.005$, ***:$p<0.0005$, ns=not significant at $\alpha=0.05$). The exact p-values for all pairwise comparisons are provided in Supplementary Data 1.

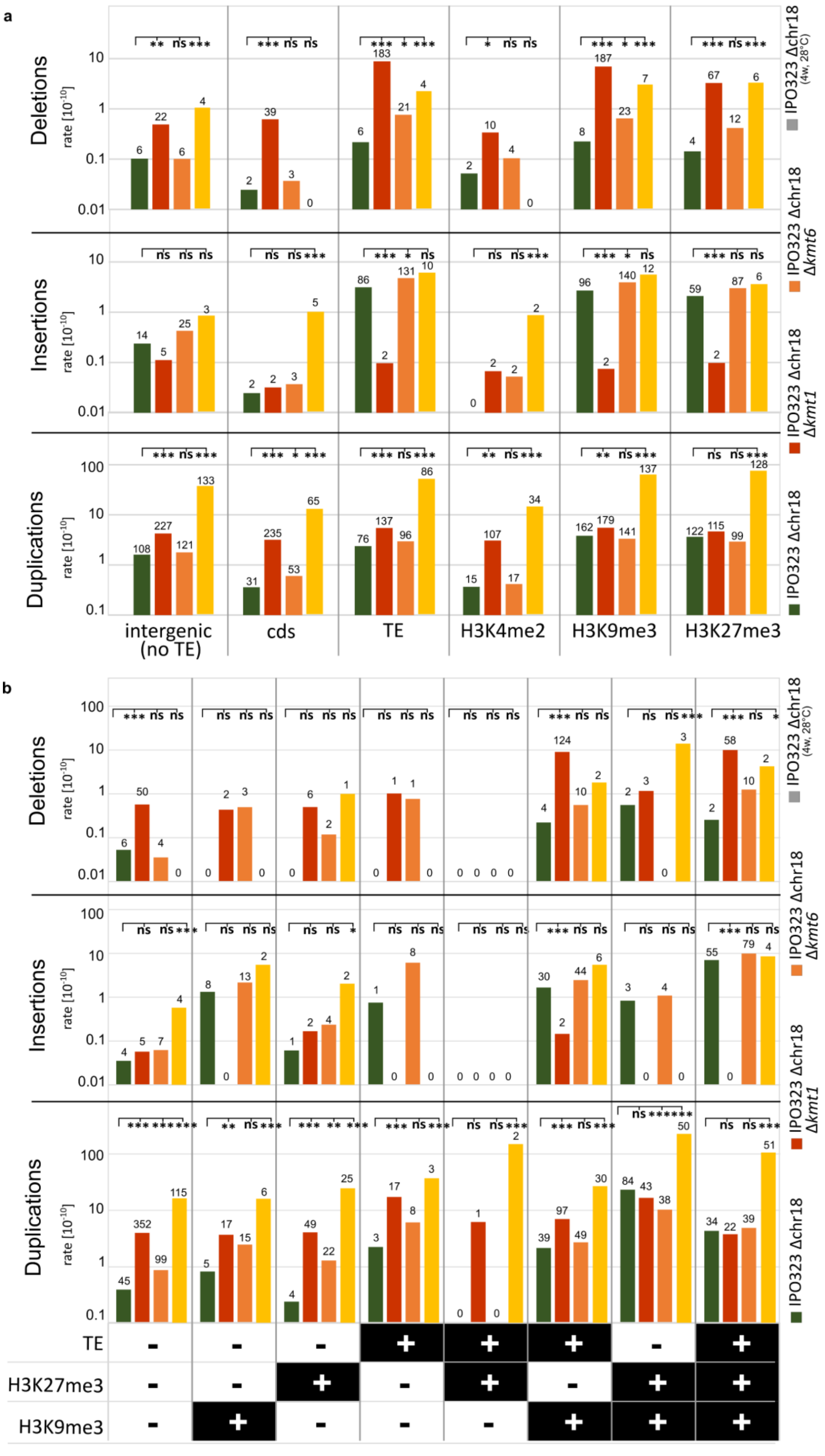
a
Deletions
Insertions
Duplication
rate [10^-10]
intergenic (no TE)
cds
TE
H3K4me2
H3K9me3
H3K27me3
IPO323 Δchr18 (4w, 28°C)
IPO323 Δchr18 Δkmt6
IPO323 Δchr18 Δkmt1
IPO323 Δchr18
b
TE
H3K27me3
H3K9me3

Supplementary Figure 6. **Frequency of occurrence the start- and end-positions of structural variants** in a) genomic compartments. b) Full factorial detailed analysis of partially features TE, H3K27me3 and H3K9me3. Bar chart depicting the rates as calculated as per site per cell division. a & b) Data was pooled for all replicates of each strain (n=40 independently evolved MA lines per strain) and are presented as mean values. Numbers above bars represent the total number of start and end-positions of the indicated structural variation located in the indicated region. Categorized p-values of FDR adjusted two-sided χ^2-test are depicted (*:p<0.05, **: p<0.005, ***: p<0.0005, ns=not significant at α=0.05). The exact p-values for all pairwise comparisons are provided in Supplementary Data 1.

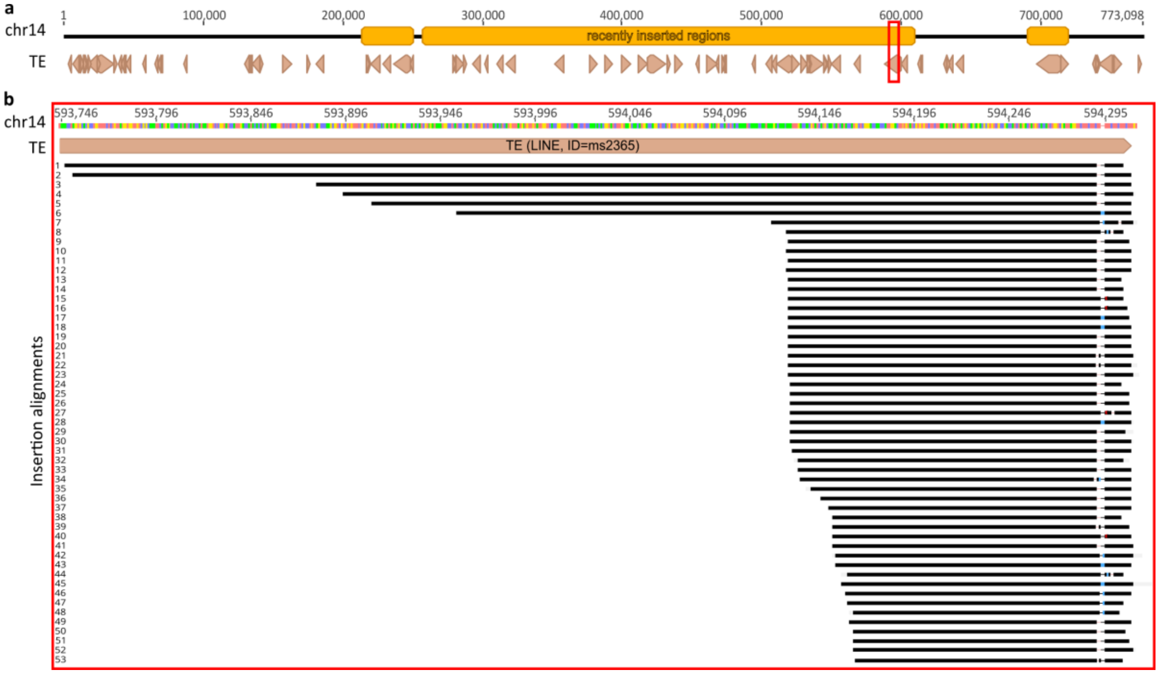

Supplementary Figure 7. **Origin of 53 independent insertions from a TE element** (LINE, ID=ms2365) in a recently inserted region of chr14. a) Overview of the chr14 with an indication of putatively inserted regions in yellow including the annotation of TEs. b) Detailed view of red box from (a) that depicts the alignment of 53 inserted sequences onto chr14:593745-594300 containing the LINE element ms2365. Black indicates sequence identity.

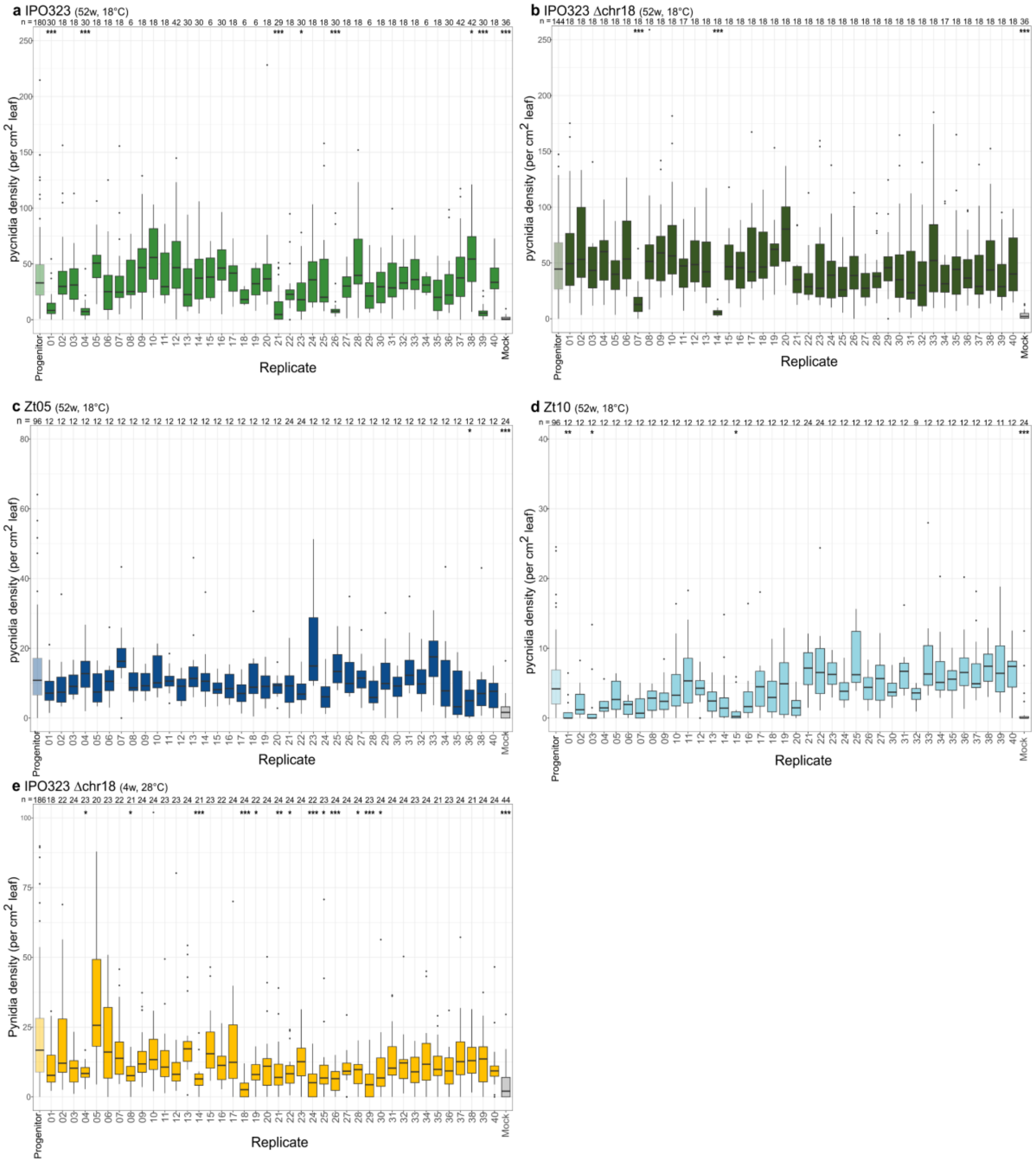

Supplementary Figure 8. **Replicated MA lines differ in their ability to produce pycnidia.** a-e) Box-whiskers plot of the observed pycnidia density per cm^2 of leaf surface for the indicated strains comparing the progenitor line with the individual replicated MA lines (n=40 independently evolved MA lines per strain). Categorized p-values of Tukey HSD on rank-based two-sided ANOVA are depicted (*: $p<0.05$, **: $p<0.005$, ***: $p<0.0005$, ns=not significant at $\alpha=0.05$). The exact p-values for all pairwise comparisons are provided in Supplementary Data 1. Box plots depict

center line, median; box limits, upper and lower quartiles; whiskers, 1.5x interquartile range; points, outliers.

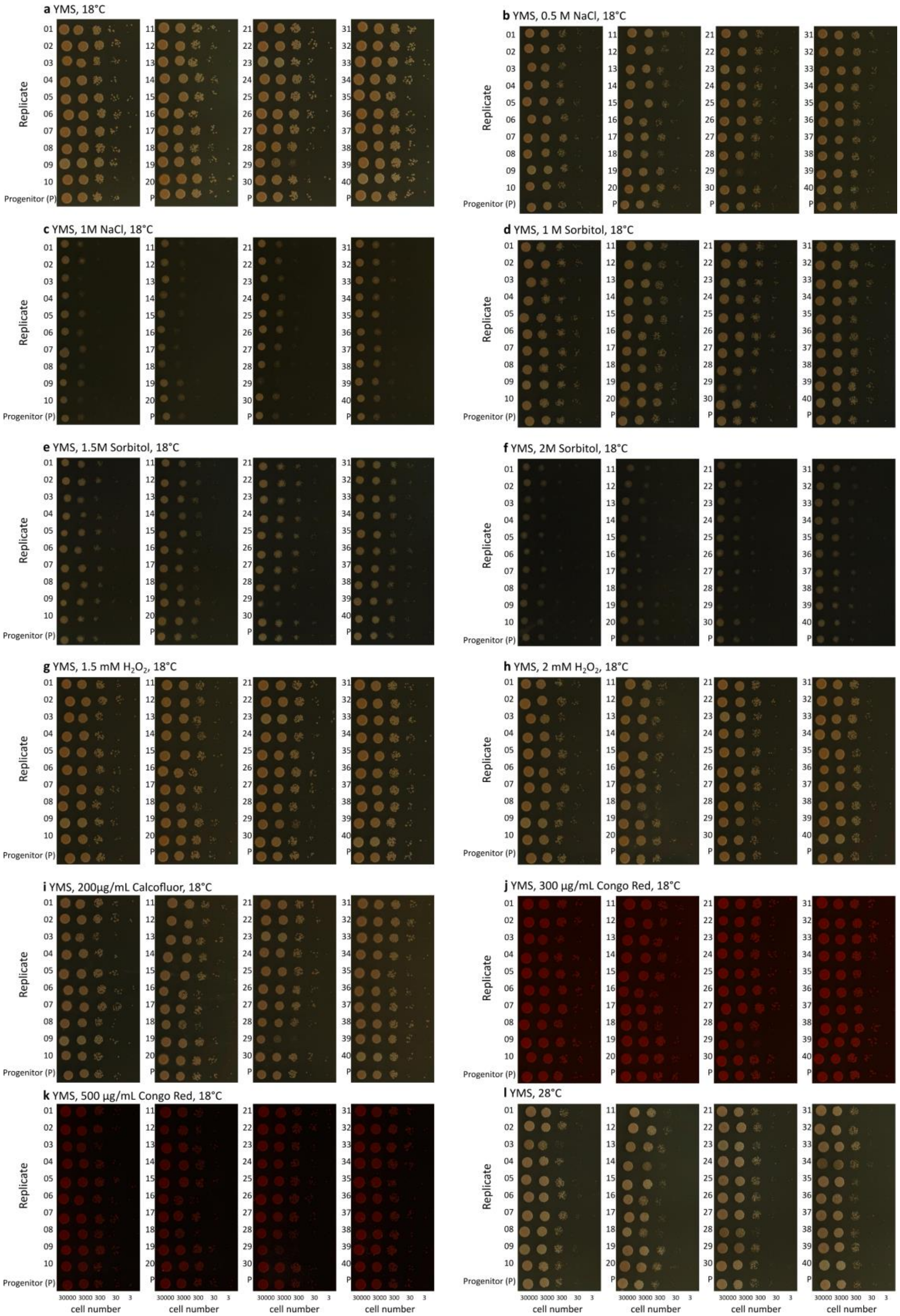

Supplementary Figure 9. **Growth phenotype of the replicated MA lines** of IPO323 ∆chr18 (4w, 28°C) in comparison to the progenitor strain under stress

conditions. a-l) The indicated number of cells was inoculated onto the indicated growth medium and growth was documented after 7 days at the specified conditions.

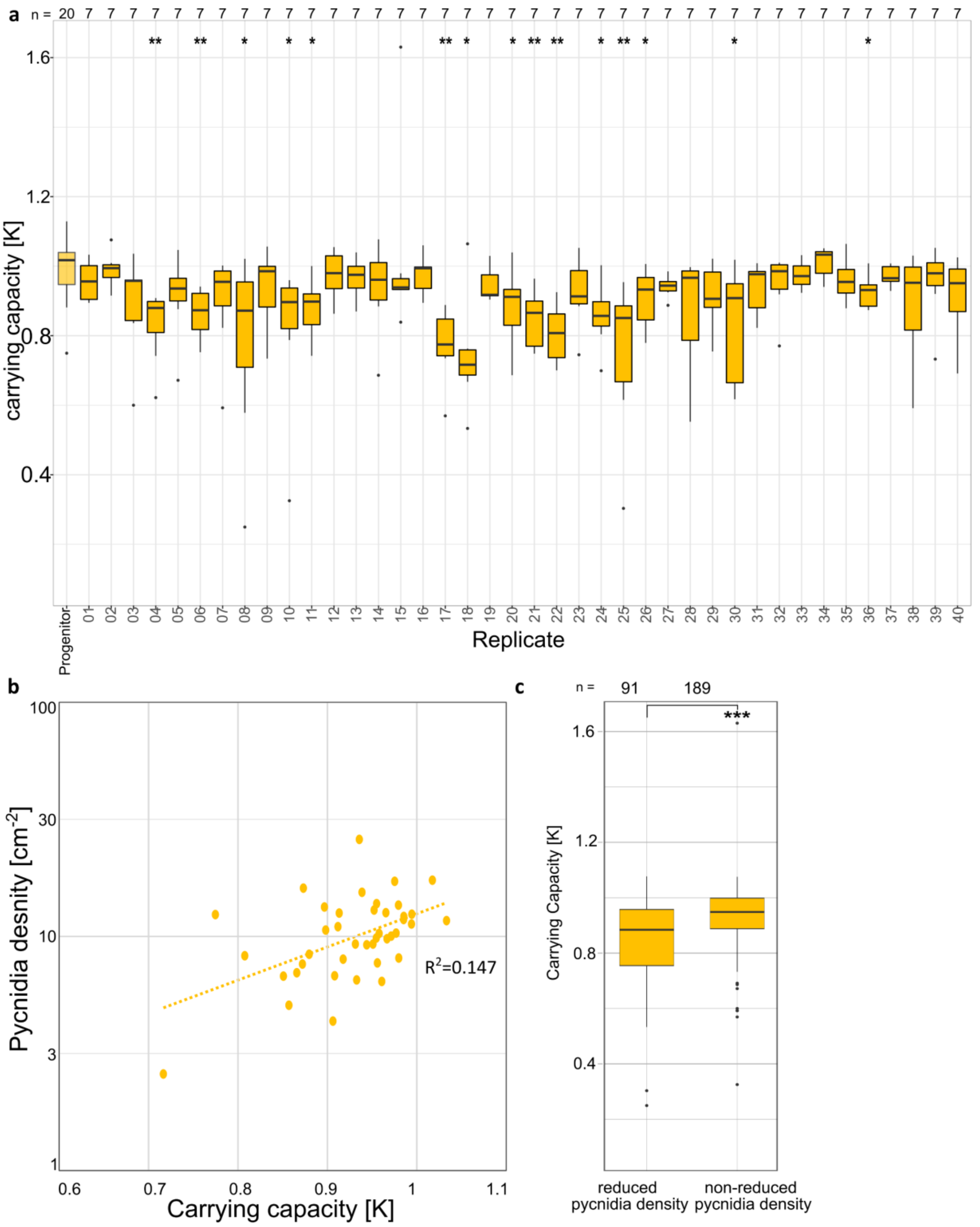

Supplementary Figure 10. **Evolved replicated MA lines differ in their carrying capacity.** a) Box-whiskers plot of the carrying capacity of replicated MA lines of IPO323 Δchr18 (4w, 28°C) in comparison to the progenitor strain b) Correlation of the mean of the carrying capacity *in vitro* and the mean of the pycnidia density *in planta*. The Pearson correlation coefficient is indicated (n=40 independently

evolved MA lines per strain). c) Box-whiskers plot of the pooled carrying capacity of replicated MA lines showing significant different pycnidia density or non-significant different pycnidia density *in planta*. Categorized p-values of FDR adjusted a) & c) Wilcoxon signed rank test are depicted (*: $p<0.05$, **: $p<0.005$, ***: $p<0.0005$, ns=not significant at $\alpha=0.05$). The exact p-values for all pairwise comparisons are provided in Supplementary Data 1.

SUPPLEMENTARY TABLES

Supplementary Table 1. **Summary table of detected novel genetic changes in the 280 replicate lines**

Strain	Number of replicates	conditions	Number of cell divisions/ line	SNPs	Small INDELs	large insertion (>10bp)	large deletion (>10bp)	duplic ations	inver sions	chromosom e/contig loss	chromosom e/contiq duplication
IPO323	40	52 weeks, 18°C	1031.9	520	388	22	20	199	3	25	5
Zt05	40	52 weeks, 18°C	919.6	440	1406	5	41	52	0	11	8
Zt10	40	52 weeks, 18°C	959.4	6791	509	1	5	25	1	4	3
IPO323 Δchr18	40	52 weeks, 18°C	1076.7	504	292	34	7	93	1	21	2
IPO323 Δchr18 Δ*kmt1*	40	52 weeks, 18°C	832.1	989	368	3	122	281	4	53	9
IPO323 Δchr18 Δ*kmt6*	40	52 weeks, 18°C	1069.3	403	419	53	15	123	2	6	6
IPO323 Δchr18 28°C, 4w	40	4 weeks, 28°C	66.3	166	195	6	4	123	1	30	15
□				9813	3577	124	214	896	12	150	48

Supplementary Table 2. **Determination of growth rates of the different strains.**

Strain	Temperatur [°C]	cell concentration [/ml] 0h	21.25h	29.25h	45.25h	54.17h	Doubling time [h]	Average of doubling time [h]	Number of duplications/52 weeks or 4 weeks (28°C only)
IPO323	18°C	1.0E+05	3.4E+06	5.7E+06	3.7E+07	7.0E+07	8.34		
IPO323	18°C	1.0E+05	3.5E+06	5.8E+06	3.2E+07	6.4E+07	8.53	8.47	1031.9
IPO323	18°C	1.0E+05	3.3E+06	6.5E+06	3.2E+07	6.3E+07	8.53		
IPO323 Δchr18	18°C	1.0E+05	3.8E+06	8.1E+06	4.6E+07	7.4E+07	8.20		
IPO323 Δchr18	18°C	1.0E+05	3.4E+06	1.0E+07	3.4E+07	9.4E+07	8.14	8.11	1076.7
IPO323 Δchr18	18°C	1.0E+05	3.3E+06	8.4E+06	4.0E+07	9.7E+07	8.00		
Zt05	18°C	1.0E+05	1.0E+06	3.8E+06	9.2E+06	3.6E+07	9.52		
Zt05	18°C	1.0E+05	1.0E+06	4.2E+06	1.0E+07	3.6E+07	9.45	9.50	919.6
Zt05	18°C	1.0E+05	9.7E+05	4.0E+06	9.6E+06	3.4E+07	9.52		
Zt10	18°C	1.0E+05	1.4E+06	3.1E+06	1.8E+07	2.7E+07	9.52		
Zt10	18°C	1.0E+05	1.4E+06	2.9E+06	1.9E+07	4.5E+07	8.88	9.11	959.4
Zt10	18°C	1.0E+05	1.4E+06	2.7E+06	1.9E+07	4.2E+07	8.91		
IPO323 Δchr18 Δkmt1	18°C	1.0E+05	1.1E+06	3.1E+06	9.2E+06	1.5E+07	10.75		
IPO323 Δchr18 Δkmt1	18°C	1.0E+05	1.1E+06	2.7E+06	8.4E+06	1.8E+07	10.56	10.50	832.1
IPO323 Δchr18 Δkmt1	18°C	1.0E+05	8.0E+05	2.9E+06	8.6E+06	2.0E+07	10.18		
IPO323 Δchr18 Δkmt6	18°C	1.0E+05	3.2E+06	7.8E+06	4.1E+07	7.6E+07	8.20		
IPO323 Δchr18 Δkmt6	18°C	1.0E+05	2.3E+06	3.6E+06	3.1E+07	7.9E+07	8.20	8.17	1069.3
IPO323 Δchr18 Δkmt6	18°C	1.0E+05	2.7E+06	8.3E+06	3.7E+07	8.5E+07	8.11		
IPO323 Δchr18	28°C	1.0E+05	9.0E+06	1.6E+07	2.9E+07	3.3E+07	9.75		
IPO323 Δchr18	28°C	1.0E+05	1.0E+07	1.6E+07	2.3E+07	2.9E+07	10.19	10.14	66.3
IPO323 Δchr18	28°C	1.0E+05	9.2E+06	1.6E+07	2.3E+07	2.3E+07	10.47		

Supplementary Table 3. **Average size of genomic compartment of the full factorial analysis of TE, H3K9me3 and H3K27me3.**

Strain	Genomic compartment: H3K9me3	H3K27me3	TE	Size [bp]*	Base substitutions
IPO323 (52w, 18°C)	-	-	-	26724157	229
	+	-	-	1402492	22
	-	+	-	3792078	65
	-	-	+	518023	13
	-	+	+	50510	1
	+	-	+	4194652	83
	+	+	-	829036	26
	+	+	+	1803872	81
IPO323 Δchr18 (52w, 18°C)	-	-	-	26383764	209
	+	-	-	1404099	12
	-	+	-	3827406	48
	-	-	+	308975	2
	-	+	+	50506	0
	+	-	+	4190237	116
	+	+	-	834728	18
	+	+	+	1815178	99
IPO323 Δchr18 Δ*kmt1* (52w, 18°C)	-	-	-	26362387	417
	+	-	-	1382070	29
	-	+	-	3598464	85
	-	-	+	294722	6
	-	+	+	48308	3
	+	-	+	4125280	236
	+	+	-	772696	34
	+	+	+	1742359	179
IPO323 Δchr18 Δ*kmt6* (52w, 18°C)	-	-	-	26378858	166
	+	-	-	1410154	13
	-	+	-	3963310	45
	-	-	+	305297	5
	-	+	+	50983	2
	+	-	+	4203740	90
	+	+	-	859197	13
	+	+	+	1862219	69
IPO323 Δchr18 (4w, 28°C)	-	-	-	26371006	29
	+	-	-	1398496	2
	-	+	-	3773313	10
	-	-	+	301092	2
	-	+	+	49656	0
	+	-	+	4172715	28
	+	+	-	814405	38
	+	+	+	1792992	57

* average of compartment size of the 40 replicated lines

Supplementary Table 4. **List of all primers used in the study**

Name	Sequence	Name	Sequence
oES3396	TCTTTCGGAGAACGGCGAAA	oES3495	GCGACATATCTGCTCCACGA
oES3397	ACACCTACCGTTTCGAGCTG	oES3508	CGAATAATAGTACCCGTACGAGTAATAGTA
oES3398	GATAACATGGGCCGAGGAGG	oES3509	GTCCTTTCGGAGGTATAGGAACTATATTAT
oES3399	TCCACACTCGTAGTCCACCA	oES3512	TGTCTTCTCCTTTGATCCTGCTATC
oES3402	AGAACGTTTGGTCAGGCGAT	oES3513	AAACACAAGCTACAACAGAACAACA
oES3403	CTGGAACACGCTCTGCAAAC	oES3514	TAGTTAACGTACGGATTACCCTTAAAGATA
oES3412	CCCGCGTGCTCAAAATCAAA	oES3515	CTTATATAACCTACTAGTCGACAACGTCTA
oES3413	GACCGTGCAAGTAGTGTGGA	oES3516	ATACGACGGTTAGAACTCTAAAGTCTATAG
oES3414	TCTATGCAAACAGCCTGCGA	oES3517	AAGAGCTAGAACAGTTAGTGGAATCTATAA
oES3415	GAAAGACGCGAGCGAACATC	oES3518	ATATGCAGCCACGGGGTATG
oES3416	CACCCACTCCATCAACCCTC	oES3519	ATGATGTGCATGGCCAAGGA
oES3417	GACTCACATCATGGGCCACA	oES3520	CGGATCCAACACAGGACACA
oES3418	GTGGTTGGGTGATACGACGT	oES3521	GATACCTCTTCTTCCCGCGG
oES3419	CTCCACCCTCCAACCCAATC	oES3522	AAACCCGTCGGAGAAGTGTC
oES3420	CGCACCATCCTTGACTGACT	oES3523	CTCCAGGTCGATCGAAGCTC
oES3421	AAAGGCATGCGCACAAGAAG	oES3526	CATCGTCGATGCTTACCCGA
oES3428	TCTAGCGCGCTCGACTTTAG	oES3527	CCAAAAGGGGTTGGGGTCTT
oES3429	CCCTCCTAATTGCGAGCGAT	oES3530	GATCAGCTTGCCGAGATCGA
oES3432	TGCTTGTCGTGGCTCATGAT	oES3531	TTAGCTCCCACACGGTGTTC
oES3433	TCGGTGTCAACAGCTGTCTC	oES3532	GCATGCCAGACATAACGCTG
oES3434	CGTGACTACCGATGTCTCCG	oES3533	TAGCGATGCTGAGGATGCAG
oES3435	CCGGGCCGTACTGTAAGATC	oES3534	ATCAAACTCCACGTCCCACC
oES3436	AGCACGAACACGAAGGACAT	oES3535	TCGAGACAGACACACAACCG
oES3437	CGCGACCTTGATGCTGAAAG	oES3536	CACTTCAAACCCACTGCTGC
oES3438	TCAAGCGTGCCAAGACATCT	oES3537	CCCTTCGCTTGGACAGTCAT
oES3439	CGTTCTCCTCCACGTGTTGA	oES3538	GGCCGAGTCTGATACGACAG
oES3440	GAGGCTCTGCGATACTCACC	oES3539	TCCTGGACGTTGCTGACTTC
oES3441	CCCAATCGACTCGATCCCTG	oES3540	GCGTCTTTTCGTGCTTGTGT
oES3442	TCGTGTTCAAGGTTGTGGCT	oES3541	CTTGAAAGACGTGTTGCGCA
oES3443	GCTAGCTCTCGAATGACGCT	oES3542	CATTTAGCAACGTCGAGGCG
oES3444	CGGATCGGCCAACTTACAGA	oES3543	TGAACAGGGAAGGCGTGATC
oES3445	GTCCTGCCGAGAACGAAAGA	oES3544	TCTTTCCTCCCGTCAGCAAC
oES3446	TTCGCTCCACTCCTGGAAAC	oES3545	GCTTTGGGTGTGCGATTGTT
oES3447	CGAGACAACCAAGTCCGGAA	oES3550	CCCTAACCCGAACTTTATTTATTAACTCTT
oES3450	TGAAAAGCGTGTTCACTGCG	oES3551	GTCTTAGTATACTAGTTAGGTTCGGAGAAA
oES3451	CGAGAGTCGGACTTGGATCG	oES3552	TCTAGTAGGTAGTATAGCAGGTAGTTTAGT
oES3452	AACTGTGTGACCACCAAGCA	oES3553	GCGGTAAACTATACAATTAGAGTAAAAGGT
oES3453	ATGGTCATGACGCTAGCTCG	oES3554	CTAATCCTAAGGCTTATATAATCGACAAGC
oES3454	TGGTAATCAGTGCTCGGCTG	oES3555	CCGTTGTTATTACTAAACAGAGTTACCTAG
oES3455	GTTCGCACAATCCTCGATGC	oES3556	TTAAGTGTCCTATCTATCAGAGCACTAATT
oES3456	GGAGGAGAGCGGTAGAGAGT	oES3557	GTTGAGATATTTAATACGGCGTAAAATGTC
oES3457	TATACTCGCGGGATAGGGCT	oES3560	GCCTTCGTTTCTATATTAACTACTATAGCC
oES3458	CACCGAACGCCCTCTTAACT	oES3561	TACGTAGTTCCGAATTCTATTCCTTTAAAC
oES3459	AGCACTATGGCGCAATCGTA	oES3562	ACGGTAGTTCTAGTAGATCTTAAAGAGAAG
oES3460	TTCGAACACCCTTAGCGACC	oES3563	CCTACTAATAGACTATCGTCGTTATCGTAA
oES3461	GTCTTGGAATTCCTCGGGCA	oES3566	CGGTGATGGTAGGCATGGTT
oES3462	TGTGGTGGAATCTGACACGG	oES3567	CTCGTCGCTTCGCGAAAAAT
oES3463	CGTCGGCTCGATTTGGAAAC	oES3568	GACCGATCACAGACGAGTCC
oES3464	TTACGGAAGCGTGGAAACGA	oES3569	GGCGAGGTAGAATTGGGCTT
oES3465	GCATTGCACACGATACGCTT	oES3570	GGTCTCCGTACCTACCTCCG
oES3466	ATGCAACGAGGATGGCTTCA	oES3571	GGGAGCCAGCGTTACTTTCT
oES3467	TCCTGGTCATTGGTGATGGC	oES3572	AGATGCCGTTAGCAGCGAAT
oES3468	CTCGTTGCGCTCGAATGAAG	oES3573	CAGCGACTCGCGACTTTAGA
oES3469	TACCGAGCAAGCAAAGTCGT	oES3580	TGTCCTAGAACCTATAGCCTACCC
oES3470	AGAATCCCGATCGCTCGTTC	oES3581	CTCACACGTAGTTGGCAGGA
oES3471	TGACAAGCTTCTCGGTGACC	oES3582	AACGCCGGCAATCCTAAAAC
oES3472	CTAAAGCCGTCCGTCGTACA	oES3583	AGGATGTCGTGCGCTAACAG
oES3473	GGGGGTAGCCCGGGATAATA	oES3586	TTGTGCTGTACAAAGCGTGC
oES3474	TCTCAAACTGCGCTGTCCAT	oES3587	CGGGGAAGTATGCGGTCTAC
oES3475	TAAAAATCGTCTGCCGCCCT	oES3588	CTGCTTGTAGGTCCTGCCTT
oES3476	AGTTCACGACAACTCGACCC	oES3589	ATCGTTCGACGTTCGGTCTC
oES3477	GAGAGGGCTTGCTGTGTGAT	oES3590	ATAGCTAAAGTCCGAGCCGC
oES3492	GAATGCGACGCTACAAGCTG	oES3591	GAGGGTAAGTCGTTAGGCCG
oES3493	CGACCAGCTCTTGTCCAGTT	oES3592	CGGGTAGGATACTCCTCGGT
oES3494	CTTGGGATTGCGGAAGGTCT	oES3593	TTCTATCCGTTTCGCCGCTT

SUPPLEMENTARY REFERENCES

1. Sambrook, J. & Russell, D. W. *Molecular Cloning. A laboratory manual.* 3rd ed. (Gold Spring Harbor Laboratory Pr, Gold Spring Harbor, New York, 2001).
2. Goodwin, S. B. *et al.* Finished Genome of the Fungal Wheat Pathogen Mycosphaerella graminicola Reveals Dispensome Structure, Chromosome Plasticity, and Stealth Pathogenesis. *PLoS Genet* **7,** e1002070; 10.1371/journal.pgen.1002070 (2011).
3. Feurtey, A. *et al.* Genome compartmentalization predates species divergence in the plant pathogen genus Zymoseptoria. *BMC genomics* **21,** 1–15 (2020).
4. Haueisen, J. *et al.* Highly flexible infection programs in a specialized wheat pathogen. *Ecology and evolution* **9,** 275–294; 10.1002/ece3.4724 (2019).
5. Bolger, A. M., Lohse, M. & Usadel, B. Trimmomatic: a flexible trimmer for Illumina sequence data. *Bioinformatics (Oxford, England)* **30,** 2114–2120; 10.1093/bioinformatics/btu170 (2014).
6. Langmead, B. & Salzberg, S. L. Fast gapped-read alignment with Bowtie 2. *Nature methods* **9,** 357–359; 10.1038/nmeth.1923 (2012).
7. Habig, M., Bahena-Garrido, S. M., Barkmann, F., Haueisen, J. & Stukenbrock, E. H. The transcription factor Zt107320 affects the dimorphic switch, growth and virulence of the fungal wheat pathogen Zymoseptoria tritici. *Mol. Plant Pathol.*; 10.1111/mpp.12886 (2019).
8. Long, H., Behringer, M. G., Williams, E., Te, R. & Lynch, M. Similar mutation rates but highly diverse mutation spectra in ascomycete and basidiomycete yeasts. *Genome Biol Evol* **8,** 3815–3821 (2016).
9. Lynch, M. & Walsh, B. *The origins of genome architecture* (Sinauer Associates Sunderland, MA, 2007).
10. Lynch, M. & Walsh, B. *Genetics and analysis of quantitative traits* (Sinauer Sunderland, MA, 1998).

11. Schotanus, K. *et al.* Histone modifications rather than the novel regional centromeres of Zymoseptoria tritici distinguish core and accessory chromosomes. *Epigenetics & chromatin* **8,** 41; 10.1186/s13072-015-0033-5 (2015).

12. Grandaubert, J., Bhattacharyya, A. & Stukenbrock, E. H. RNA-seq based gene annotation and comparative genomics of four fungal grass pathogens in the genus Zymoseptoria identify novel orphan genes and species-specific invasions of transposable elements. *G3 (Bethesda, Md.),* g3. 115.017731 (2015).

13. Layer, R. M., Chiang, C., Quinlan, A. R. & Hall, I. M. LUMPY. A probabilistic framework for structural variant discovery. *Genome biology* **15,** R84 (2014).

14. Chiang, C. *et al.* SpeedSeq. Ultra-fast personal genome analysis and interpretation. *Nature methods* **12,** 966–968; 10.1038/nmeth.3505 (2015).

15. Zhang, J., Kobert, K., Flouri, T. & Stamatakis, A. PEAR. A fast and accurate Illumina Paired-End reAd mergeR. *Bioinformatics (Oxford, England)* **30,** 614–620; 10.1093/bioinformatics/btt593 (2014).

16. Bankevich, A. *et al.* SPAdes: a new genome assembly algorithm and its applications to single-cell sequencing. *Journal of computational biology : a journal of computational molecular cell biology* **19,** 455–477; 10.1089/cmb.2012.0021 (2012).

17. Huang, W., Li, L., Myers, J. R. & Marth, G. T. ART. A next-generation sequencing read simulator. *Bioinformatics (Oxford, England)* **28,** 593–594; 10.1093/bioinformatics/btr708 (2012).
