# Supplementary material for: Epigenetic modifications affect the rate of spontaneous mutations in a pathogenic fungus
Source: Nat Commun. 2021 Oct 7;12:5869. doi: 10.1038/s41467-021-26108-y (PMC8497519; doi:10.1038/s41467-021-26108-y)
Supplement: Supplementary file 3 — Description of Additional Supplementary Files [file 41467_2021_26108_MOESM3_ESM.pdf]

Description of Additional Supplementary Files

File Name: Supplementary Data 1
Description: Description and results of all statistical tests and source data

File Name: Supplementary Data 2
Description: Summary of sequencing and assembly statistics

File Name: Supplementary Data 3
Description: All in planta phenotypic data

File Name: Supplementary Data 4
Description: Validation of randomly chosen mutations using Sanger sequencing

File Name: Supplementary Data 5
Description: List of all novel mutations in replicates compared to progenitor

File Name: Supplementary Data 6
Description: Results of simulation for the detection of structural variations using an assembly-based approach

File Name: Supplementary Data 7
Description: Gene annotation of reference isolate IPO323

File Name: Supplementary Data 8
Description: TE annotation of reference isolate IPO323

File Name: Supplementary Data 9
Description: H3K4me2 enriched regions of reference isolate IPO323Δchr18

File Name: Supplementary Data 10
Description: H3K9me3 enriched regions of reference isolate IPO323Δchr18

File Name: Supplementary Data 11
Description: H3K27me2 enriched regions of reference isolate IPO323Δchr18
