# Supplementary material for: Epigenetic modifications affect the rate of spontaneous mutations in a pathogenic fungus
Source: Nat Commun. 2021 Oct 7;12:5869. doi: 10.1038/s41467-021-26108-y (PMC8497519; doi:10.1038/s41467-021-26108-y)
Supplement: Supplementary file 15 — Reporting Summary [file 41467_2021_26108_MOESM15_ESM.pdf]

nature portfolio

Corresponding author(s): Michael Habig
Eva H. Stukenbrock

Last updated by author(s): Sep 14, 2021

Reporting Summary

Nature Portfolio wishes to improve the reproducibility of the work that we publish. This form provides structure for consistency and transparency in reporting. For further information on Nature Portfolio policies, see our Editorial Policies and the Editorial Policy Checklist.

Statistics

For all statistical analyses, confirm that the following items are present in the figure legend, table legend, main text, or Methods section.

n/a	Confirmed	
☐	☒	The exact sample size (*n*) for each experimental group/condition, given as a discrete number and unit of measurement
☐	☒	A statement on whether measurements were taken from distinct samples or whether the same sample was measured repeatedly
☐	☒	The statistical test(s) used AND whether they are one- or two-sided<br>*Only common tests should be described solely by name; describe more complex techniques in the Methods section.*
☒	☐	A description of all covariates tested
☐	☒	A description of any assumptions or corrections, such as tests of normality and adjustment for multiple comparisons
☐	☒	A full description of the statistical parameters including central tendency (e.g. means) or other basic estimates (e.g. regression coefficient) AND variation (e.g. standard deviation) or associated estimates of uncertainty (e.g. confidence intervals)
☐	☒	For null hypothesis testing, the test statistic (e.g. *F*, *t*, *r*) with confidence intervals, effect sizes, degrees of freedom and *P* value noted<br>*Give P values as exact values whenever suitable.*
☒	☐	For Bayesian analysis, information on the choice of priors and Markov chain Monte Carlo settings
☒	☐	For hierarchical and complex designs, identification of the appropriate level for tests and full reporting of outcomes
☐	☒	Estimates of effect sizes (e.g. Cohen's *d*, Pearson's *r*), indicating how they were calculated

Our web collection on statistics for biologists contains articles on many of the points above.

Software and code

Policy information about availability of computer code

Data collection	see supplementary Information
Data analysis	Trimmomatic (V 0.38)<br>Bowtie2 (V 2.3.5)<br>samtools (V 1.7)<br>Picard (V 2.18.20)<br>bcftools (V 1.6)<br>R (V R3.6.0)<br>R Studio (V 1.2.1335)<br>growthrates (V 0.8.2)<br>SpeedSeq (V 0.1.2)<br>PEAR (V 0.9.11)<br>SPADES (V 3.13.1)<br>ART (V 2.5.8)

For manuscripts utilizing custom algorithms or software that are central to the research but not yet described in published literature, software must be made available to editors and reviewers. We strongly encourage code deposition in a community repository (e.g. GitHub). See the Nature Portfolio guidelines for submitting code & software for further information.

Data

Policy information about availability of data

All manuscripts must include a data availability statement. This statement should provide the following information, where applicable:

- Accession codes, unique identifiers, or web links for publicly available datasets
- A description of any restrictions on data availability
- For clinical datasets or third party data, please ensure that the statement adheres to our policy

Sequencing reads have been deposited in the Sequence Read Archive and are available under the BioProject PRJNA614493 https://www.ncbi.nlm.nih.gov/bioproject/?term=PRJNA614493 and PRJNA718981 https://www.ncbi.nlm.nih.gov/bioproject/PRJNA718981/ The genome sequence of the reference isolate IPO323 used in this study is available under the accession GCA_000219625.1 at the European Nucleotide Archive https://www.ebi.ac.uk/ena/browser/view/GCA_000219625.1?show=blobtoolkit. Supplementary Data 1-11 including the IPO323 gene annotations, regions of histone modification enrichment and TE annotations and source data are available at DOI 10.5281/zenodo.5413239. The assembled genomes of Zt05 and Zt10 and the respective annotations are available at https://zenodo.org/record/3820378

Field-specific reporting

Please select the one below that is the best fit for your research. If you are not sure, read the appropriate sections before making your selection.

☒ Life sciences ☐ Behavioural & social sciences ☐ Ecological, evolutionary & environmental sciences

For a reference copy of the document with all sections, see nature.com/documents/nr-reporting-summary-flat.pdf

Life sciences study design

All studies must disclose on these points even when the disclosure is negative.

Sample size	Sample size was choosen similar to previously published mutation accumulation studies (e.g. Dillon, M. M., Sung, W., Sebra, R., Lynch, M. & Cooper, V. S. Genome-wide biases in the rate and molecular spectrum of spontaneous mutations in Vibrio cholerae and Vibrio fischeri. Mol. Biol. Evol. 34, 93–109 (2017). and Long, H., Behringer, M. G., Williams, E., Te, R. & Lynch, M. Similar mutation rates but highly diverse mutation spectra in ascomycete and basidiomycete yeasts. Genome Biol Evol 8, 3815–3821 (2016).)
Data exclusions	No data was excluded from the study
Replication	The mutation accumulation experiment comprised 40 replicated independently evolved lines for each genome x environment condition. All quantitative phenotypical assessments were replicated at least three times and all data were included in the analysis.
Randomization	All phenotypical assessments of all treatments were randomized.
Blinding	All phenotypical assessments of all treatments were blinded to the operator

Reporting for specific materials, systems and methods

We require information from authors about some types of materials, experimental systems and methods used in many studies. Here, indicate whether each material, system or method listed is relevant to your study. If you are not sure if a list item applies to your research, read the appropriate section before selecting a response.

Materials & experimental systems

n/a	Involved in the study	
☒	☐	Antibodies
☒	☐	Eukaryotic cell lines
☒	☐	Palaeontology and archaeology
☒	☐	Animals and other organisms
☒	☐	Human research participants
☒	☐	Clinical data
☒	☐	Dual use research of concern

Methods

n/a	Involved in the study	
☒	☐	ChIP-seq
☒	☐	Flow cytometry
☒	☐	MRI-based neuroimaging
